# Some population parameters of three flatfish species (*Arnoglossus laterna*, *Monochirus hispidus*, and *Microchirus ocellatus*) from Güllük Bay, southern Aegean Sea

**DOI:** 10.7717/peerj.21227

**Published:** 2026-05-18

**Authors:** Hasan Cerim, Sercan Yapıcı, Özgen Yılmaz, Anıl Gülşahin, Ozan Soykan, İsmail Reis, Ferhat Yalgın

**Affiliations:** 1 Faculty of Fisheries, Muğla Sıtkı Koçman University, Muğla, Turkey; 2 Alaca Avni Çelik Vocational School, Hitit University, Çorum, Turkey; 3 Faculty of Fisheries, Ege University, İzmir, Turkey; 4 Underwater Technologies, Bandırma Onyedi Eylül University, Balıkesir, Turkey

**Keywords:** Flatfish, Von Bertalanffy growth, Length–weight relationship, Demersal species, Aegean Sea

## Abstract

This study investigates the age, growth, and length–weight relationships of three demersal flatfish species—*Monochirus hispidus*, *Microchirus ocellatus*, and *Arnoglossus laterna*—collected from Güllük Bay in the southern Aegean Sea between September 2019 and February 2020. A total of 667 individuals were analyzed to determine biometric characteristics and growth parameters. The relationship between length and weight was expressed by the equation W = *a*TL^*b*^, revealing species-specific growth patterns. *A. laterna* exhibited positive allometric growth with *b* values between 3.25 and 3.35, *Monochirus ocellatus* showed slightly positive allometry with b values ranging from 3.09 to 3.19, while *Microchirus hispidus* displayed nearly isometric growth with *b* values around 3.03. The sex ratio of *A. laterna* was significantly male-biased, whereas *Monochirus hispidus* and *Microchirus ocellatus* showed balanced distributions. The estimated von Bertalanffy Growth parameters produced the following growth equations: *A. laterna* as L_t_ = 21.3[1–e^−0.21(t+0.62)^], *Monochirus hispidus* as L_t_ = 14.7[1–e^−0.38(t+0.04)^], and *Microchirus ocellatus* as L_t_ = 17.56[1–e^−0.22(t+1.57)^]. These results indicate that *Monochirus hispidus* grows more rapidly but attains a smaller maximum size, whereas *A. laterna* and *Microchirus ocellatus* grow more slowly and reach larger asymptotic lengths. The findings provide valuable insights into the growth dynamics of these benthic species and highlight that studies on their age and growth are still very limited, emphasizing the need for further research.

## Introduction

Flatfishes of the order Pleuronectiformes are represented by 14 families, encompassing a total of 822 species (Gibson et al., 2015). According to FAO data, in 2023, a total of 577,092 tons of 101 flatfish species were harvested worldwide (FAO, 2025). This highlights the economic importance of many flatfish species and indicates that their living resources are heavily exploited by fisheries. For instance, STECF Scientific Technical Economic Committee for Fisheries (2017) reported that annual revenues from *Solea solea* and *Pleuronectes platessa* fisheries amount to approximately 100 million and 120 million Euros, respectively. In addition to capture fisheries, some flatfish species are also farmed, including *Paralichthys olivaceus* (*e.g.*, Stieglitz et al., 2021), *Solea senegalensis* (*e.g.*, Pinto, Engrola & Conceicao, 2018), *Scophthalmus maximus* (*e.g.*, Şahin, 2001), *Hippoglossus hippoglossus* (*e.g.*, Gallardo et al., 2022), and *Cynoglossus semilaevis* (*e.g.*, Wang et al., 2025).

*Monochirus hispidus* (whiskered sole), *Microchirus ocellatus* (foureyed sole), and *A. laterna* (Mediterranean scaldfish) are demersal flatfish species widely distributed in the Mediterranean Sea, surrounding waters, and the Eastern Atlantic. These species typically inhabit sandy and muddy bottoms at depths ranging from 10 to 300 m and feed on benthic invertebrates. Maximum lengths range between 21 and 25  cm, while maturity sizes vary among species: *Monochirus hispidus* matures at approximately 8.5  cm, whereas *Microchirus ocellatus* and *A. laterna* mature at 12–15  cm. As small benthic predators, these species serve as intermediate trophic links and play a key role in maintaining benthic energy flow. They are often caught as by-catch in commercial fisheries and can be used as ecological indicators to assess benthic community structure. According to the IUCN Red List, *Monochirus hispidus* and *A. laterna* are classified as Least Concern (LC), while *Microchirus ocellatus* is listed as Data Deficient (DD) (Froese & Pauly, 2025a; Froese & Pauly, 2025b; Froese & Pauly, 2025c).

According to FAO (2025), *A. laterna*, *Monochirus hispidus* and *Microchirus ocellatus* are also economically valuable species subject to commercial fishing. Official capture statistics reported to the FAO reveal a clear contrast in the exploitation patterns of *Microchirus ocellatus* sole, *A. laterna*, and *Monochirus hispidus* in the Mediterranean and adjacent Atlantic areas. The FAO data indicates that *A. laterna* constitutes the most commercially relevant species among the three, with annual landings in some years reaching several hundred tons—generally ranging between approximately 200 and 600 tons—primarily reported by Italy and Spain. In contrast, *Microchirus ocellatus* has historically exhibited negligible catches, with most years reporting no landings and only sporadic recent records typically remaining below 5 tons, almost exclusively from Spain. *Monochirus hispidus* displays similarly low and irregular exploitation levels, with annual landings usually fluctuating between 0 and about 10 tons, mainly contributed by Spain and Italy. Collectively, these patterns suggest that while *A. laterna* maintains moderate regional fishery importance, *Microchirus ocellatus* and *Monochirus hispidus* represent minor components of demersal fisheries and are likely encountered primarily as incidental bycatch rather than as dedicated target species.

Several studies have investigated the age, growth, and length–weight relationships of these species in different parts of the Mediterranean and adjacent waters. For *A. laterna*, age and growth parameters have been reported from the western and central Mediterranean, as well as from the Aegean and Marmara Sea (Deniel, 1990; Özütok & Avşar, 2002; Veiga et al., 2009; Bök et al., 2011; Ilkyaz et al., 2017). In contrast, studies on *Monochirus hispidus* are limited and mostly restricted to specific Mediterranean sub-regions, with reported maximum ages and growth parameters differing among areas (Stergiou, 1991; Amaral & Cabral, 2004; Karachle & Stergiou, 2008; Özekinci et al., 2009; Cengiz, 2022). For *Microchirus ocellatus*, available biological information is scarce, and age-based growth studies are virtually absent, with most existing data limited to basic morphometric descriptions or length-weight relationships (Morey et al., 2003; Özekinci et al., 2009; Mehanna & Farouk, 2021; Cengiz, 2022; Rodríguez-García et al., 2023). Despite these efforts, comprehensive and region-specific information from the Eastern Mediterranean, particularly from Turkish coastal waters, remains limited.

While these flatfish species play key ecological and economic roles, information on their age and growth is insufficient both globally and within Turkish waters. Including the present study, a total of eight age and growth studies have been conducted on *A. laterna*, *Monochirus hispidus* and *Microchirus ocellatus* primarily from Turkish waters (Özütok & Avşar, 2002; Uckun Ilhan et al., 2007; Bayhan, Sever & Taşkavak, 2008b; UckunIlhan et al., 2010; Ilkyaz et al., 2017; present study—with three species). While length–weight relationship (LWR) information for *A. laterna* is well documented in Turkish waters (*e.g.*, Mater & Bayhan, 2000; Çakır et al., 2003; Karakulak, Erk & Bilgin, 2006; Özaydın & Taskavak, 2006; Sangun, Akamca & Akar, 2007; Bayhan, Sever & Taşkavak, 2008a; Ilkyaz et al., 2008; Keskin & Gaygusuz, 2010; Bök et al., 2011; Bilge et al., 2014), comparable data for *Monochirus hispidus* and particularly *Microchirus ocellatus* remain scarce and geographically limited (Özekinci et al., 2009; Ilkyaz et al., 2008; Cengiz, 2022; present study). Understanding their population dynamics is crucial for evaluating the ecological impacts of fisheries on benthic communities. Therefore, the present study aims to determine the age, growth parameters, and length–weight relationships of *Monochirus hispidus*, *Microchirus ocellatus*, and *A. laterna* collected from the Güllük Bay, Southern Aegean Sea. The data obtained will contribute to the knowledge of the life-history traits of these species and provide baseline information for future fisheries management and ecological assessments.

## Materials and Methods

### Study area

**Figure 1:**
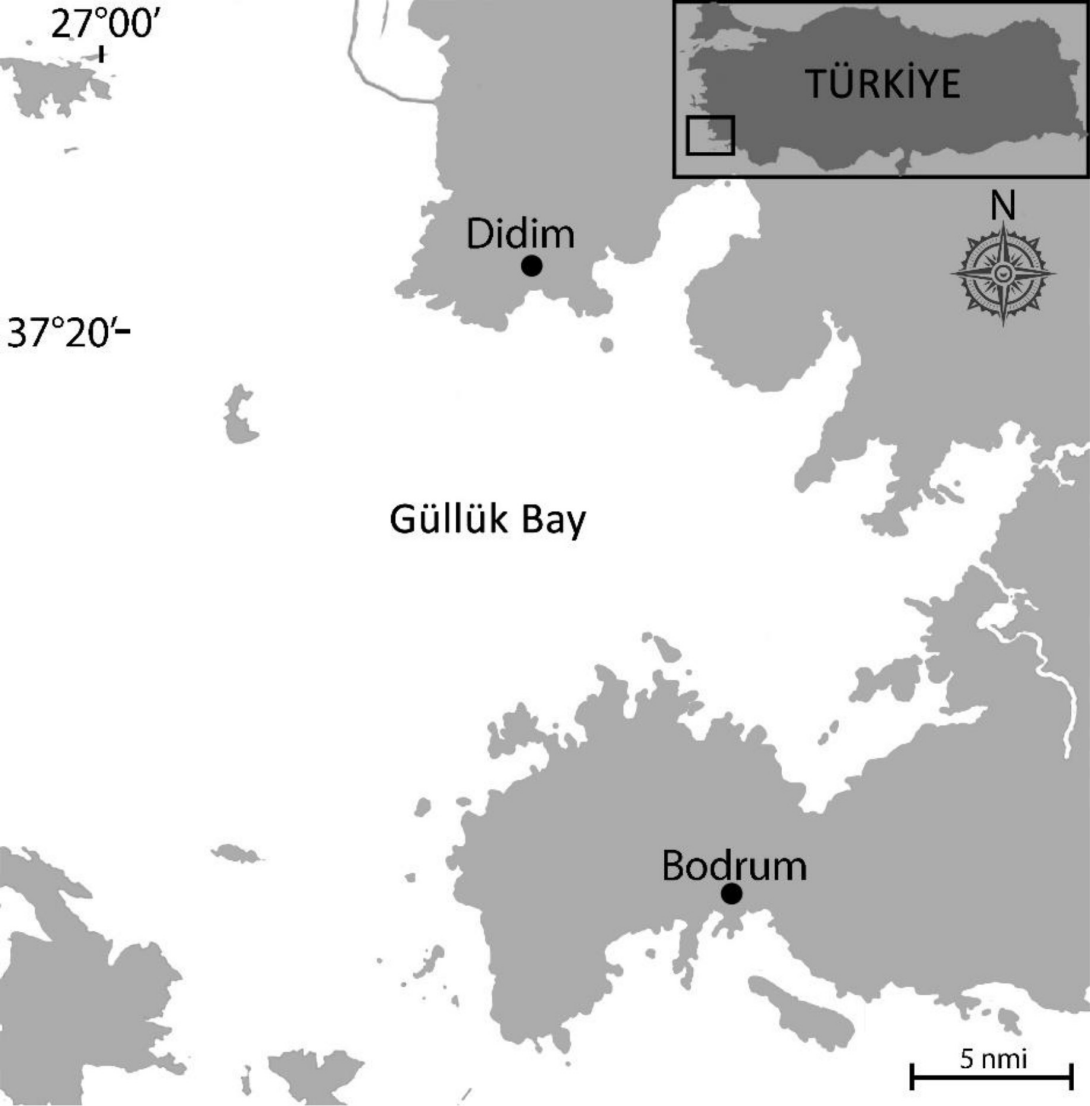
Study area.

The study was conducted using a commercial demersal trawl vessel within the legal fishing zone of the Turkish coast between September 2019 and February 2020 in Güllük Bay, southern Aegean Sea (Fig. 1). Specimens were collected using a traditional trawl net with a 44 mm cod-end mesh size. Each haul lasted three hours, with the vessel maintaining a speed of 2.5 knots. Samples were stored on ice for further laboratory examination. This study did not involve the use of live animals in experimental procedures. Specimens were collected as dead individuals from commercial trawl operations, in accordance with ethical standards for the use of fisheries bycatch; therefore, no institutional ethical approval was required.

### Laboratory examinations

The total length (TL) of the fish was measured using measurement boards (±0.1 cm) and weighed to the nearest 0.01 g. After dissection, the sex of each specimen was determined macroscopically. Females were identified by their long, red-orange gonads, while males had small, lentil-shaped, light-colored gonads. However, the sex of some individuals could not be identified due to damage to internal organs or lack of maturity.

For age estimation, vertebrae were removed from each individual and boiled to eliminate adhering soft tissues following Soykan, Gülşahin & Cerim (2020). Cleaned vertebral centra were examined under a stereo microscope using reflected light. Age readings were conducted independently by three experienced readers.

**Figure 2:**
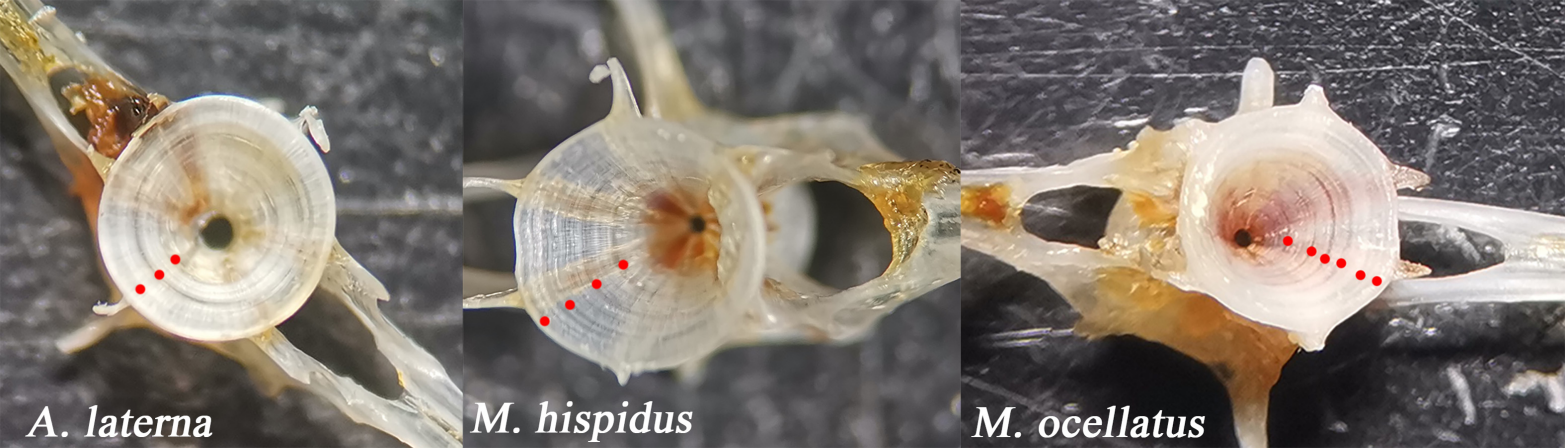
Representative vertebrae used for age determination of *A. laterna*, *Monochirus hispidus*, and *Microchirus ocellatus*. Red dots indicate the identified annuli (opaque and translucent growth bands) counted for age estimation. Distinct and regularly spaced growth increments are visible in all three species.

Age was determined based on the count of alternating opaque and translucent growth bands (annuli) from the focus to the outer margin of the centrum. The growth increments were distinct and regularly spaced, allowing consistent identification across specimens. Representative vertebrae of each species are presented in Fig. 2, with annuli indicated for clarity.

### Data analysis

Ricker’s (1973) formula, W = *a* TL^*b*^, was used to determine the relationship between length and weight. Logarithmic transformation was applied to linearize the equation; log W = log*a* + *b* logTL. Where; W; weight, TL; total length, *a*; intercept and *b*; slope of the linear regression. The significance of the b values for the species was tested using Pauly’s *t*-test, with *b* =3 indicating isometric growth.

Von Bertalanffy growth equation was fitted to size-at-age data. The function L_t_ = L_∞_[1−exp^−*K*(*t*−*t*0)^] was applied to data, where L_t_ is the fish length (cm) at time t (in year), L_∞_ is the asymptotic length (cm), K is the growth coefficient (year^−1^), and t_0_ (year) is the hypothetical time when the length is equal to zero.

Following parameters were estimated by the formulas below;

The growth performance index; *ϕ*′ = log*K* + 2log*L*∞ (Pauly & Munro, 1984)

The natural mortality; $M=1.066{L}_{\infty }^{-0.1172}{K}^{0.5092}$ (Djabali et al., 1994)

A compilation of species-specific information from the literature has been included in the Supplementary Material.

## Results

**Figure 3:**
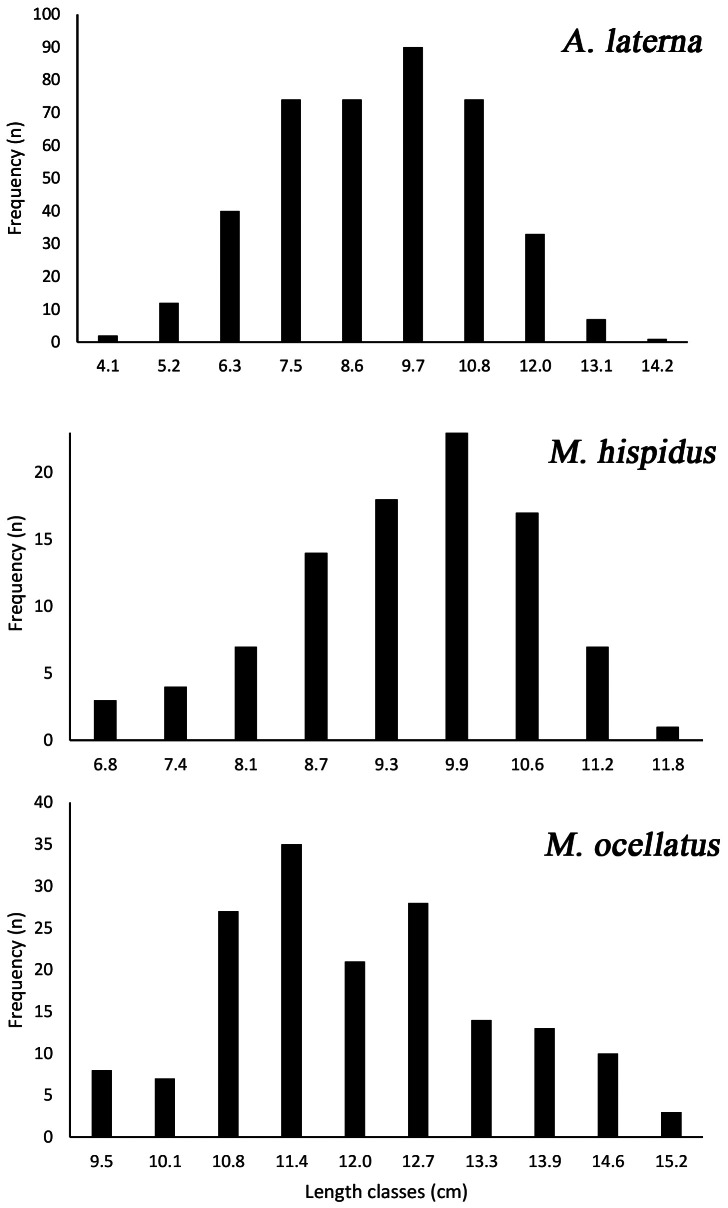
Length–frequency distributions of three flatfish species, *A. laterna*, *Monochirus hispidus*, and *Microchirus ocellatus*, collected from Turkish coastal waters of the Aegean Sea.

The length–frequency distributions of the three flatfish species are presented in Fig. 3.

For *A. laterna*, total lengths ranged from 4.1 to 14.2 cm, with the majority of individuals concentrated between approximately 7.5 and 11.0 cm. Very small and very large individuals were comparatively rare. In *Monochirus hispidus*, lengths ranged from 6.8 to 11.8 cm. The relatively narrow size range suggests a more homogeneous size structure within the sampled individuals. For *Microchirus ocellatus*, total lengths varied between 9.5 and 15.2 cm. The highest frequencies observed between 11.0 and 13.0 cm. Larger individuals (>14 cm) were present but less frequent. Overall, all three species displayed predominantly unimodal length distributions, consistent with typical demersal trawl samples where intermediate size classes are more frequently represented.

A total of 407 individuals of *A. laterna* (116 females and 178 males) were analyzed, with the allometric coefficient (*b*) estimated at 3.353 for females (*p* < 0.05), 3.314 for males (*p* < 0.05), and 3.256 for combined sexes (*p* < 0.05), indicating a positive allometric growth pattern. *Monochirus hispidus* comprised 94 individuals (49 females and 45 males), with b values ranging from 3.031 to 3.089 (*p* > 0.05 for all), suggesting isometric to slightly positive allometric growth. For *Microchirus ocellatus*, a total of 166 individuals (76 females and 90 males) were examined. The estimated *b* values were 3.181 for females (*p* < 0.05), 3.092 for males (*p* > 0.05), and 3.192 for combined sexes (*p* < 0.05). Accordingly, females and the combined sample exhibited positive allometric growth, whereas males showed isometric growth, as the *b* value did not differ significantly from 3. These results demonstrate that while all three species exhibit growth patterns ranging from isometric to positive allometry, *A. laterna* and *Microchirus ocellatus* generally gain weight faster than length as they grow, whereas *Monochirus hispidus* shows a more proportional weight-to-length increase (Table 1).

**Table 1:** Length-weight relationships of *Monochirus hispidus*, *Microchirus ocellatus*, and *A. laterna* from eastern Mediterranean.

**Species**	**Sex**	**N**	**TL**_**min**_ **–TL**_**max**_ ** (cm)**	**W**_**min**_ **–W**_**max**_ ** (g)**	***a***	***b***	**CI of** ***b***	**SE of** ***b***	**R** ^**2**^	**Growth**
*A. laterna*	F	116	5.7–13.5	1.48–28.33	0.004	3.353	3.242–3.465	0.056	0.969	A+
	M	178	6–14.2	1.74–28.8	0.0043	3.314	3.215–3.414	0.050	0.961	A+
	C	407	4.1–14.2	0.68–28.8	0.005	3.256	3.195–3.317	0.031	0.964	A+
*Monochirus hispidus*	F	49	7.9–11.4	6.9–20.83	0.0102	3.066	2.670–3.462	0.197	0.838	0
	M	45	6.8–11.8	3.41–17.7	0.0108	3.031	2.736–3.325	0.146	0.909	0
	C	94	6.8–11.8	3.41–20.83	0.0096	3.089	2.876–3.301	0.107	0.901	0
*Microchirus ocellatus*	F	76	9.7–15.2	13.9–71.42	0.0105	3.181	2.941–3.422	0.121	0.904	A+
	M	90	9.5–15.2	11.76–67.44	0.0123	3.092	2.869–3.316	0.113	0.895	0
	C	166	9.5–15.2	11.76–71.42	0.0099	3.192	3.027–3.357	0.084	0.899	A+

A total of 407 individuals of *A. laterna* were analyzed, including 116 females and 178 males. A *t*-test indicated a significant difference between the number of females and males (*p* < 0.05). For *Monochirus hispidus* (49 females and 45 males) and *Microchirus ocellatus* (76 females and 90 males), no significant differences were observed between sexes (*p* > 0.05 for both). These results suggest that *A. laterna* exhibits a skewed sex ratio favoring males, whereas *Monochirus hispidus* and *Microchirus ocellatus* populations have balanced sex distributions.

The von Bertalanffy growth parameters of the three species were determined (Table 2). For *A. laterna*, the growth coefficient (K) was estimated as 0.21, the asymptotic length (L_∞_) as 21.3 cm, and the theoretical age (t_0_) as −0.62. The natural mortality rate (M) was calculated as 0.34, and the growth performance index (*ϕ*) as 1.98. For *Monochirus hispidus*, K was 0.38, L_∞_ 14.7 cm, t_0_ −0.04, M 0.48, and *ϕ* 1.91 and *Microchirus ocellatus*, the K value was 0.22, L_∞_ 17.56 cm, t_0_ −1.57, M 0.35, and *ϕ* 1.83.

**Figure 4:**
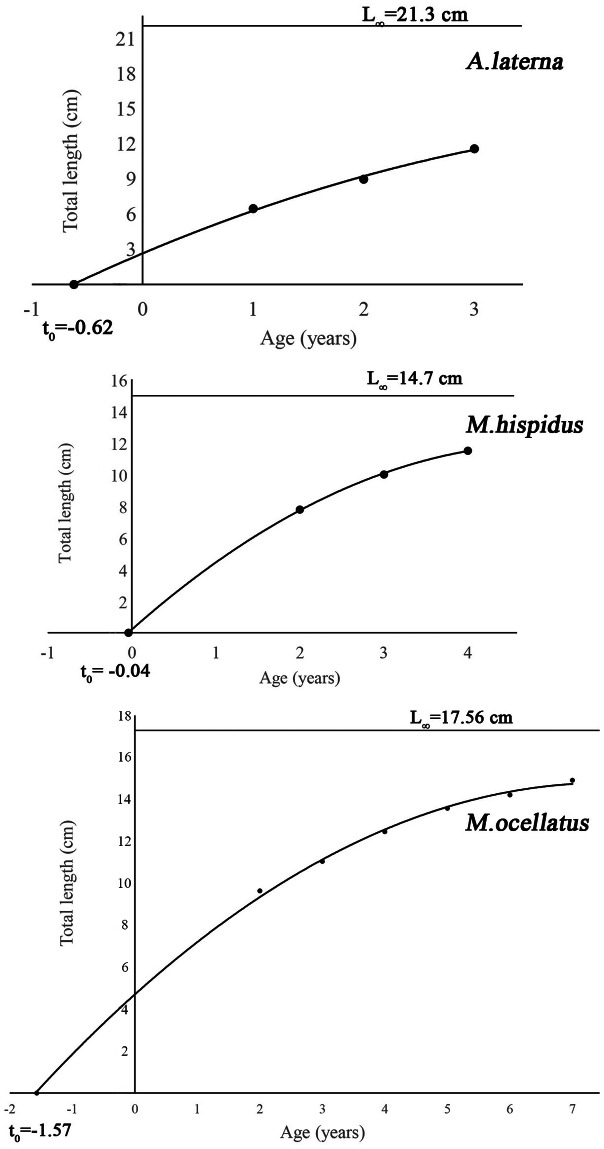
The von Bertalanffy growth curve with maximum and minimum length of *A. laterna*, *Monochirus hispidus* and *Microchirus ocellatus* from Aegean Sea.

The von Bertalanffy growth curves for *A. laterna*, *Monochirus hispidus*, and *Microchirus ocellatus* are presented in Fig. 4. Each curve illustrates the relationship between total length and age based on the estimated growth parameters. For *A. laterna*, growth was relatively slow, reaching an asymptotic length (L_∞_) of 21.3 cm at a theoretical age (t_0_) of −0.62. The model indicates a gradual increase in length with age, suggesting a species that attains a relatively large maximum size but grows at a moderate rate. *Monochirus hispidus* exhibited the highest growth coefficient among the three species, with L_∞_ = 14.7 cm and t_0_ = −0.04, reflecting a faster growth pattern and earlier attainment of asymptotic size. In contrast, *Microchirus ocellatus* showed L_∞_ = 17.56 cm and t_0_ = −1.57 years, indicating a slower early growth phase but a prolonged growth period. The extended curve pattern suggests a species that maintains growth over a wider age range compared to the others.

**Table 2:** The von Bertalanffy growth parameters, growth performance index values and natural mortality of *Monochirus hispidus*, *Microchirus ocellatus*, and *A. laterna* from eastern Mediterranean.

**Species**	**K**	**L**_∞_	**t**_**0**_	**M**	*φ*
*A. laterna*	0.21	21.3	−0.62	0.34	1.98
*Monochirus hispidus*	0.38	14.7	−0.04	0.48	1.91
*Microchirus ocellatus*	0.22	17.56	−1.57	0.35	1.83

## Discussion

### Length–weight relationships

The length–weight relationship (LWR) results obtained in the present study for *A. laterna* indicated positive allometric growth for females (*b* = 3.353), males (*b* = 3.314), and combined sexes (*b* = 3.256), showing that these fish gain weight slightly faster than length during growth. These findings are broadly consistent with previous Mediterranean studies. For instance, Matta (1959) in Italy and Giovanardi & Piccinetti (1983) in the Adriatic Sea reported *b* values ranging from 3.030 to 3.534, while Pereda & Villamor (1991) in the Bay of Biscay and Merella et al. (1997) in the Balearic Islands found *b* values between 3.389 and 3.45. In the Aegean and Eastern Mediterranean, earlier research reported slightly lower b values, ranging from 2.747 to 3.301 (Mater & Bayhan, 2000; Özütok & Avşar, 2002; Çakır et al., 2003; Uckun Ilhan et al., 2007; Bayhan, Sever & Taşkavak, 2008b; Ilkyaz et al., 2008). The slight differences in *b* values across regions may reflect local environmental conditions, prey availability, or differences in sampling methodology. High R^2^ values (0.96–0.97) in the present study indicate a strong correlation between length and weight, confirming the reliability of the measurements. Overall, the current results align with historical data but suggest subtle regional variation in growth patterns, highlighting the importance of localized studies for fisheries assessment and management.

The length–weight relationship (LWR) of *Monochirus hispidus* in the present study revealed an overall positive allometric growth, with b values ranging from 3.031 to 3.089, indicating that the species gains weight slightly faster than length as it grows. These results are generally higher than the earlier studies from the northern Aegean region, where unsexed individuals showed *b* values of 2.43–2.64 (Karachle & Stergiou, 2008; Özekinci et al., 2009; Cengiz, 2022). The observed differences may be attributed to regional variations in environmental conditions, food availability, and sampling sizes, as well as the methodological differences in measuring and analyzing specimens. The present study exhibited high R^2^ values (0.838–0.909), suggesting a strong correlation between length and weight, consistent with the robustness reported in previous works. Overall, while the LWR patterns of *Monochirus hispidus* align with earlier Mediterranean studies, the slightly higher *b* values observed in the current dataset may reflect localized growth conditions or temporal changes in population dynamics.

The length–weight relationship (LWR) of *Microchirus ocellatus* in the present study showed that individuals ranged from 9.5 to 15.2 cm, with allometric coefficients (*b*) of 3.181 for females, 3.092 for males, and 3.192 for the combined population, indicating a positive allometric growth pattern. When compared to previous studies, our *b* values are slightly higher. For instance, Mehanna & Farouk (2021) reported *b* = 2.461 for individuals 6–21 cm long in the Eastern Mediterranean, Abdallah (2002) found *b* = 2.9 for 9.3–16.6 cm specimens off Alexandria, and Cengiz (2022) recorded *b* = 2.7 for 10.2–13.5 cm fish from the Gallipoli Peninsula. Additionally, Rodríguez-García et al. (2023) found *b* = 3.007 for 5.2–15.4 cm individuals in the Gulf of Cadiz, while Ilkyaz et al. (2008) reported *b* = 3.25 for 7.7–12.7 cm fish in Izmir Bay. The present study’s R^2^ values (0.895–0.904) and relatively narrow confidence intervals indicate high reliability of the measurements. Overall, these findings suggest that *Microchirus ocellatus* in the Southern Aegean Sea exhibits slightly stronger positive allometric growth compared to populations studied elsewhere, reflecting faster weight gain relative to length within the observed size range (Mehanna & Farouk, 2021; Abdallah, 2002; Cengiz, 2022; Rodríguez-García et al., 2023; Ilkyaz et al., 2008; Morey et al., 2003; Özekinci et al., 2009).

### Age and growth patterns

The growth parameters of *A. laterna* exhibit noticeable regional and sex-based variations. In the Adriatic Sea, Giovanardi & Piccinetti (1983) reported an asymptotic length (L_∞_) of 15.8 cm with a growth coefficient (K) of 0.57 year^−^^1^ and t_0_ of −0.773 years for combined sexes. In contrast, populations from the Aegean Sea show more variable values: Özütok & Avşar (2002) found L_∞_ values ranging between 14.55–15.88 cm, K between 0.13–0.15 year^−^^1^, and t_0_ between −1.54 and −1 year, indicating slower growth and an earlier theoretical age at length zero compared to the Adriatic population. Subsequent studies in İzmir Bay (Uckun Ilhan et al., 2007; Uckun Ilhan et al., 2010; Bayhan, Sever & Taşkavak, 2008b) reported slightly higher L_∞_ values (16.34–17.58 cm) with K values between 0.236–0.412 year^−^^1^, reflecting faster growth rates, while t_0_ ranged from −0.379 to −0.887 years. The most recent data from the east-central Aegean Sea (Ilkyaz et al., 2017) indicated an L_∞_ of 20.62 cm and K of 0.245 year^−^^1^, with t_0_ at −1.071 years, suggesting that populations in this region may reach larger maximum sizes but grow more slowly. In comparison, the present study recorded an L_∞_ of 21.3 cm, K of 0.21 year^−^^1^, and t_0_ of −0.62 years. These values fall within the range reported for the Aegean Sea and indicate a moderate growth rate coupled with a relatively higher asymptotic length compared to most earlier regional estimates. Overall, these differences likely reflect the influence of local environmental conditions, fishing pressure, and sampling strategies on the growth dynamics of *A. laterna* (Giovanardi & Piccinetti, 1983; Deniel, 1990; Özütok & Avşar, 2002; Uckun Ilhan et al., 2007; Uckun Ilhan et al., 2010; Bayhan, Sever & Taşkavak, 2008b; Ilkyaz et al., 2017).

The growth parameters of *Monochirus hispidus* vary among the studied regions. In the Eastern Mediterranean, males exhibited a growth coefficient (K) of 0.411 y^−^^1^ with an asymptotic length (L_∞_) of 11.2 cm, while females showed slightly lower growth (K = 0.378 y^−^^1^; L_∞_ = 14.8 cm) (Stergiou, 1991). In Italy (Sardinia), K and t_0_ values were similar to those observed in Greece, with males having K = 0.411 y^−^^1^ and t_0_ = −0.94 years, and females K = 0.378 y^−^^1^ and t_0_ = −0.03 years (Cau & Deiana, 1983). Populations from Portugal showed markedly lower growth rates for females (K = 0.24 y^−^^1^, t_0_ = −1.13 years) but higher for males (K = 0.7 y^−^^1^, t_0_ = −0.25 years), suggesting potential environmental or local adaptation effects on growth dynamics (Amaral & Cabral, 2004).

This study provides the first estimates of growth and mortality parameters for *Microchirus ocellatus*, establishing a fundamental baseline for understanding the species’ life history. The von Bertalanffy growth parameters (L_∞_ = 17.56 cm TL, *K* = 0.22 yr^−^^1^, t_0_ = −1.57 years) reveal a growth strategy characterized by a moderate maximum size and a relatively slow growth rate. The combination of these values suggests that *Microchirus ocellatus* adopts a conservative life-history strategy, likely allocating energy towards long-term survival and periodic reproduction rather than rapid somatic growth. As the first study of its kind, these parameters are critical for future ecological comparisons and for assessing how this species may respond to environmental changes or anthropogenic pressures.

### Natural mortality and growth performance

In certain studies, natural mortality (M) values were not originally reported; however, based on the available biological parameters provided in those studies, M was estimated in the present work using the method proposed by Djabali et al. (1994), and subsequent interpretations were made accordingly.

The natural mortality (M) values for *A. laterna*, calculated according to Djabali et al. (1994), vary across regions and populations. In the Adriatic Sea, Giovanardi & Piccinetti (1983) reported an M of 0.6 year^−^^1^, while in France, Deniel (1990) found slightly higher M values of 0.79 year^−^^1^ for combined sexes. Turkish populations exhibit lower natural mortality estimates; Özütok & Avşar (2002) reported M values ranging from 0.26–0.29 year^−^^1^ in the Yumurtalık Bight, and subsequent studies in İzmir Bay (Uckun Ilhan et al., 2007; Uckun Ilhan et al., 2010; Bayhan, Sever & Taşkavak, 2008b) showed M values between 0.36 and 0.53 year^−^^1^. The most recent east-central Aegean population studied by Ilkyaz et al. (2017) had an M of 0.36 year^−^^1^, indicating moderate mortality compared to earlier studies. These results suggest that natural mortality is generally lower in Turkish Aegean populations than in northern Mediterranean populations, potentially reflecting differences in environmental conditions, predation pressure, and fishing intensity (Giovanardi & Piccinetti, 1983; Deniel, 1990; Özütok & Avşar, 2002; Uckun Ilhan et al., 2007; Uckun Ilhan et al., 2010; Bayhan, Sever & Taşkavak, 2008b; Ilkyaz et al., 2017).

The growth performance index (*ϕ*′) of *A. laterna* shows notable regional variation. In the Adriatic Sea, Giovanardi & Piccinetti (1983) reported a *ϕ*′ of 2.15, whereas French populations studied by Deniel (1990) had slightly higher values, with *ϕ*′ reaching 2.38 for males and 2.32 for females. Turkish populations display a broader range: in the Yumurtalık Bight, Özütok & Avşar (2002) calculated *ϕ*′ values around 1.50–1.55, while in İzmir Bay, Uckun Ilhan et al. (2007), Uckun Ilhan et al. (2010) and Bayhan, Sever & Taşkavak (2008b) reported *ϕ*′ values between 1.50 and 2.17, indicating relatively faster growth in some local populations. The east-central Aegean population studied by Ilkyaz et al. (2017) exhibited a *ϕ*′ of 2.02, and the present Southern Aegean study showed a higher *ϕ*′ of 1.79 (Present Study). Overall, these *ϕ*′ values suggest that growth performance varies geographically, with northern Mediterranean populations generally exhibiting higher growth indices than southern populations, potentially due to differences in temperature, productivity, and ecological conditions (Giovanardi & Piccinetti, 1983; Deniel, 1990; Özütok & Avşar, 2002; Uckun Ilhan et al., 2007; Uckun Ilhan et al., 2010; Bayhan, Sever & Taşkavak, 2008b; Ilkyaz et al., 2017).

The natural mortality rate (*M* = 0.48 year^−^^1^) calculated for the *Monochirus hispidus* population in Güllük Bay shows significant geographic variability when compared with values reported for other populations across the Mediterranean basin. Our findings are highly consistent with those reported from Greek waters (*M* = 0.47−0.51) (Stergiou, 1991) and Sardinia, Italy (*M* = 0.47−0.51) (Cau & Deiana, 1983). This consistency suggests that *Monochirus hispidus* populations in the Central and Eastern Mediterranean are subject to similar life-history strategies and environmental pressures. However, the values reported for the population in the Sado Estuary, Portugal, show a marked divergence from the Mediterranean populations. Amaral & Cabral (2004) reported a wider range of M for this population, varying between 0.37 and 0.67. The lower M value (0.37), in particular, may indicate that the estuarine environment, with its sheltered nature and potentially different food web dynamics, affords the population a lower natural mortality rate. In contrast, the higher M value (0.67) reported in the same study could reflect annual fluctuations or varying pressures on different age classes within this population. This situation clearly demonstrates that natural mortality rates can vary significantly not only between species but also among populations of the same species inhabiting different habitats.

The life expectancy (Ø’ = 1.91) calculated in our study is consistent with the values reported from other Mediterranean populations (1.71–1.92). This supports the notion that individuals in the Mediterranean share a similar lifespan profile. Furthermore, it can be suggested that there is no fundamental difference in life expectancy between the Atlantic and Mediterranean populations of this species.

The natural mortality rate (*M* = 0.35 yr^−^^1^) and the overall growth performance index (Ø’ = 1.83) reported here represent the first quantitative assessment of population dynamics for this species. The moderate mortality rate aligns with the observed slow growth, painting a picture of a population with a balanced energy budget between growth and maintenance. In the absence of prior age-based studies, these results cannot be placed in a regional or temporal context. Therefore, this study does not merely present data; it establishes the essential foundational framework upon which all future research on the age, growth, and population dynamics of *Microchirus ocellatus* will be built. Subsequent studies will now be able to use these values as a reference point to investigate spatial variability, temporal trends, and the impacts of fishing on this species.

### The ecological significance of size (length) distributions across different age classes

The age-length distributions and von Bertalanffy growth parameters presented in this study reveal distinct life-history strategies among the three sympatric flatfish species—*A. laterna*, *Monochirus hispidus*, and *Microchirus ocellatus*—which have significant ecological implications for their coexistence and population dynamics.

The estimated asymptotic length (L∞) shows a clear gradient: *A. laterna* (L_∞_ = 21.3 cm) >*Microchirus ocellatus* (L_∞_ = 17.56 cm) >*Monochirus hispidus* (L_∞_ = 14.7 cm). This variation in maximum potential size reflects a niche partitioning mechanism, reducing direct competition for resources among the three species. *A. laterna* likely exploits a different trophic niche or habitat zone compared to the other two species.

The interspecific variation in growth parameters highlights the need for species-specific management strategies in multispecies fisheries. The differential growth rates and maximum sizes imply varying vulnerabilities to fishing pressure and environmental changes. *Monochirus hispidus*, with the smallest asymptotic size, may be particularly vulnerable to size-selective fishing mortality and require more conservative management measures.

Understanding these species-specific growth trajectories and population structures is essential for predicting how these flatfish species will respond to climate change, habitat modification, and fishing pressure, thereby informing ecosystem-based fisheries management approaches in the region.

### Sex ratio

The analysis of sex ratios revealed distinct interspecific patterns among the studied flatfish species. While the populations of *Monochirus hispidus* and *Microchirus ocellatus* exhibited a balanced sex distribution, a statistically significant skew towards males was observed in *A. laterna* (*p* < 0.05). This disparity in *A. laterna* could be attributed to several ecological and biological factors. Sex-specific variations in mortality rates are commonly reported in both freshwater and marine fish species, which are often attributed to differences between sexes in age at maturity, growth rates, or behavioral patterns, particularly during the spawning period (Bunnell et al., 2012). Furthermore, environmental parameters, particularly temperature during larval development, can significantly influence sex determination in many teleost species, potentially leading to skewed sex ratios in wild populations (Ospina-Álvarez & Piferrer, 2008). Although not directly measured in this study, differential spatial distribution or habitat use between sexes could also lead to sampling biases in trawl surveys (Loher, 2011). In contrast, the balanced sex ratios observed in *Monochirus hispidus* and *Microchirus ocellatus* suggest a stable population structure, which is a positive indicator for their reproductive potential and long-term population viability. Future research integrating genetic sex identification with detailed studies of spawning behavior and habitat preference is recommended to elucidate the precise mechanisms underlying the skewed sex ratio in

The near 1:1 sex ratio observed in *Monochirus hispidus* and *Microchirus ocellatus* is a strong indicator of stable and sustainable population structures for these species. A balanced ratio maximizes reproductive potential by increasing the likelihood of mate availability for every individual, thereby enhancing recruitment success and maintaining genetic diversity within the population (Schmickl & Karsai, 2010). This stability suggests that these species are well-adapted to the current habitat conditions and are not under intense sex-specific selective pressures or sex-dependent mortality.

### Non-target species status and ecological significance as bio-indicators

Although *A. laterna*, *Monochirus hispidus*, and *Microchirus ocellatus* appear in FAO fishery records, the minimal reported catches strongly suggest they are taken as by-catch rather than as target species. The flatfish species are common components of by-catch in bottom trawl fisheries targeting high-value species like shrimp, sole, or hake (Kelleher, 2005). Their capture and subsequent discarding can have significant consequences. Discard mortality is often high for flatfish due to the physical trauma experienced during trawl capture and sorting on deck, leading to unaccounted fishing mortality that can impact their populations (Broadhurst, Suuronen & Hulme, 2006). This unregulated mortality poses a threat to the long-term sustainability of these non-target species and can alter the structure and function of benthic communities by selectively removing a group of mid-level predators.

Due to their specific life-history characteristics and strong benthic–pelagic coupling, flatfishes (Order Pleuronectiformes) may serve as valuable bio-indicators of ecosystem condition. Shifts in their life-history traits and size structure may potentially indicate trawling disturbance and chronic fishing pressure on benthic habitats. Likewise, trophic metrics derived from their feeding ecology may reflect alterations in food-web structure. Furthermore, contaminant loads accumulated in their tissues, together with associated pathological responses, could provide insight into environmental pollution and ecosystem health degradation.

### Fisheries and management implications

The von Bertalanffy growth parameters—asymptotic length (L_∞_) and growth coefficient (K)—are key determinants of a stock’s intrinsic productivity (King & McFarlane, 2003). In our study, the three flatfish species exhibited distinct growth strategies that directly influence their population dynamics and vulnerability to fishing pressure.

*Monochirus hispidus* displayed the highest growth coefficient (*K* = 0.38 year^−^^1^) among the three species, indicating rapid growth toward its relatively small asymptotic length (L_∞_ = 14.7 cm). This life history strategy typically allows for faster population turnover and potentially greater resilience to fishing mortality, as individuals reach harvestable sizes quickly and the population can replace itself more rapidly (King & McFarlane, 2003).

In contrast, *A. laterna* and *Microchirus ocellatus* exhibited slower growth patterns (*K* = 0.21 and 0.22 year^−^^1^, respectively) but attained larger maximum sizes (L_∞_ = 21.3 and 17.56 cm, respectively). Species with this combination of slower growth and larger maximum size are generally more vulnerable to overexploitation (Jennings, Reynolds & Mills, 1998). Their slower growth rates mean that it takes longer for individuals to reach sizes vulnerable to fishing gears, and populations require more time to recover from fishing pressure. This is particularly concerning for *A. laterna*, which also showed a male-biased sex ratio—an additional factor that may further impact reproductive potential and population resilience.

These interspecific differences in growth strategies have important implications for fisheries management in Güllük Bay. The faster-growing *Monochirus hispidus* may sustain higher fishing pressure compared to the slower-growing *A. laterna* and *Microchirus ocellatus*, which would require more conservative management approaches to prevent overexploitation.

### A foundation for data-limited management

This study demonstrates that for data-poor fisheries, fundamental life-history parameters such as natural mortality (M) and von Bertalanffy growth parameters (L_∞_ and K) are not merely useful information but the essential foundation for management.

The growth and mortality parameters presented in this study are not merely descriptive statistics; they are the essential building blocks for calculating sustainable fishing limits, designing selective fishing gears, and ultimately ensuring the long-term viability of these flatfish populations and the ecosystems they inhabit.

For many marine species, particularly non-target and by-catch species like *A. laterna*, *Monochirus hispidus*, and *Microchirus ocellatus*, the lack of robust biological and fisheries data creates significant uncertainties that severely challenge effective and sustainable management. This data deficiency permeates all aspects of the management cycle, may lead to decisions made in an environment of high risk.

### The precautionary approach and the burden of proof

The global analysis by Costello et al. (2016) reveals that the prevailing “business-as-usual” fishery management effectively operates with a reversed burden of proof, where fishing continues largely unchecked until stocks are severely depleted. Their modeling demonstrates that this reactive approach projects a future where widespread collapse becomes the norm, with 88% of stocks projected to be unhealthy by 2050. However, the study provides powerful evidence that shifting to a proactive, rights-based management strategy can rapidly reverse this trajectory, rebuilding the median fishery in just a decade and simultaneously increasing catch, profits, and biomass, thereby proving that prevention is vastly superior to seeking a cure after the fact. The Precautionary Approach, a cornerstone of modern fisheries management, is difficult to implement without basic data to define what constitutes a “precautionary” level of fishing.

### Limitations and future research

While this study provides crucial baseline data on the life history parameters of *A. laterna*, *Monochirus hispidus*, and *Microchirus ocellatus*, several limitations should be considered when interpreting the results and for guiding future research.

First, the study was conducted within a specific geographic region—the Aegean Sea coast of Türkiye—which restricts the direct extrapolation of results to other parts of the Mediterranean or adjacent seas. Geographic variation in environmental conditions and fishing pressure can lead to significant differences in the life-history traits of fish populations, as evidenced by studies such as Audzijonyte et al. (2020) and Baudron et al. (2014), which highlight the complex and often localized responses of fish to warming and exploitation. Second, the temporal scope and sampling resolution may influence the generalizability of the results. As emphasized by Hilborn et al. (2020), environmental regimes and climate change fundamentally constrain stock productivity and recruitment, introducing uncertainty into management targets and recovery timelines. Long-term monitoring is essential to fully understand population responses to both environmental and anthropogenic drivers. Finally, sample sizes for certain species and demographic groups—such as *Monochirus hispidus*, with a total of 94 individuals—while adequate for initial parameter estimation, may limit the statistical power and precision required for more complex analyses. Future studies would benefit from expanded sampling, particularly for rare species or specific sex and age classes, to support more robust and detailed life-history comparisons.

Despite these limitations, this study successfully establishes a critical foundation of knowledge for these previously data-poor species. The identified constraints clearly outline the path for future research, emphasizing the need for long-term, spatially expansive, and ecosystem-oriented studies.

## Conclusion

The critical data gaps in age and growth parameters for many marine species, including the flatfish species studied here, represent a significant impediment to both scientific understanding and effective fisheries governance. Filling these gaps is not merely an academic exercise but a fundamental prerequisite for sustainable marine resource management. In conclusion, the investment in filling age and growth data gaps is an investment in the future sustainability of fisheries and the health of marine ecosystems. The data presented in this study for *A. laterna*, *Monochirus hispidus*, and *Microchirus ocellatus* represent a significant step forward. However, a concerted, ongoing effort is required to expand this knowledge base geographically and temporally, transforming the management of data-poor species from an exercise in guesswork into a science-based practice.

##  Supplemental Information

10.7717/peerj.21227/supp-1 Supplemental Information 1 Raw data

## Additional Information and Declarations

### Field Study Permissions

The following information was supplied relating to field study approvals (*i.e.*, approving body and any reference numbers): Not applicable. No specific field permits were required for this study, as all specimens were obtained as by-catch from legally operating commercial fisheries in Güllük Bay.

### Data Availability

The following information was supplied regarding data availability: The raw data is available in the Supplemental File.
